# Neural basis of hierarchical visual form processing of Japanese *Kanji* characters

**DOI:** 10.1002/brb3.413

**Published:** 2015-11-04

**Authors:** Hiroki Higuchi, Yoshiya Moriguchi, Hiroki Murakami, Ruri Katsunuma, Kazuo Mishima, Akira Uno

**Affiliations:** ^1^ Graduate School of Comprehensive Human SciencesUniversity of TsukubaLaboratory of Advanced ResearchD 1‐1‐1 TennodaiTsukubaIbaraki305‐8577Japan; ^2^ Japan Society for the Promotion of Science (JSPS) Research FellowKojimachi Business Center Building5‐3‐1 KojimachiChiyodaTokyo102‐0083Japan; ^3^ Department of PsychophysiologyNational Institute of Mental HealthNational Center of Neurology and Psychiatry4‐1‐1 OgawahigashiKodairaTokyo187‐0031Japan; ^4^ Integrative Brain Imaging CenterNational Center of Neurology and Psychiatry4‐1‐1 OgawahigashiKodairaTokyo187‐0031Japan; ^5^ Department of PsychologyNagoya UniversityFuro‐choChikusa‐kuNagoyaAichi464‐8601Japan; ^6^ Faculty of Human SciencesUniversity of TsukubaLaboratory of Advanced ResearchD 1‐1‐1 TennodaiTsukubaIbaraki305‐8577Japan

**Keywords:** Hierarchical coding, Japanese logographic *Kanji*, *Kanji* radical, Left occipitotemporal cortex, visual character form

## Abstract

**Introduction:**

We investigated the neural processing of reading Japanese *Kanji* characters, which involves unique hierarchical visual processing, including the recognition of visual components specific to *Kanji*, such as “radicals.”

**Methods:**

We performed functional MRI to measure brain activity in response to hierarchical visual stimuli containing (1) real *Kanji* characters (complete structure with semantic information), (2) pseudo *Kanji* characters (subcomponents without complete character structure), (3) artificial characters (character fragments), and (4) checkerboard (simple photic stimuli).

**Results:**

As we expected, the peaks of the activation in response to different stimulus types were aligned within the left occipitotemporal visual region along the posterior–anterior axis in order of the structural complexity of the stimuli, from fragments (3) to complete characters (1). Moreover, only the real *Kanji* characters produced functional connectivity between the left inferotemporal area and the language area (left inferior frontal triangularis), while pseudo *Kanji* characters induced connectivity between the left inferotemporal area and the bilateral cerebellum and left putamen.

**Conclusions:**

Visual processing of Japanese *Kanji* takes place in the left occipitotemporal cortex, with a clear hierarchy within the region such that the neural activation differentiates the elements in *Kanji* characters' fragments, subcomponents, and semantics, with different patterns of connectivity to remote regions among the elements.

## Introduction

When we read words, we initially process the words in our brain as a form of visual information. Recent studies have focused on clarifying the neural basis specific to the visual processing of word forms, and neuropsychological and neuroimaging studies have demonstrated the involvement of the left occipitotemporal cortex in the ventral visual stream. For example, patients with pure alexia, which is linked with damage to the left occipitotemporal cortex, manifest letter‐by‐letter reading (Warrington and Shallice 1980; Arguin and Bub 1993). In addition, a number of neuroimaging studies have demonstrated activation of a patch of cortex to the fusiform gyrus in word and letter reading tasks but not in object recognition tasks (e.g., Dehaene et al. 2004; Szwed et al. 2011). Other researchers have shown that split brain patients show reading performance equivalent to that of healthy controls, with appropriate left fusiform gyrus activation, only when words are presented to their right visual field (Cohen et al. 2000). The evidence suggests that the left (and not the right) fusiform gyrus specializes in word form processing, and Cohen and colleagues labeled this region the “visual word form area” (VWFA).

How is visual word form information processed within the occipitotemporal cortex? If we consider the visual information not restricted to words (e.g., general objects), numerous neuroimaging studies have been performed. According to the evidence, simpler visual features (e.g., dots) activate the more posterior part of the occipitotemporal cortex, whereas more complicated patterns (e.g., geometric figures) activate the anterior part of the inferior temporal regions (Grill‐Spector et al. 1998; Lerner et al. 2001; Kamitani and Tong 2005; Dumoulin and Hess 2007). This suggests that visual information is processed through the occipitotemporal cortex from posterior to anterior, in a sequential manner according to its complexity. Visual word forms are supposed to be processed in the same manner in the ventral visual stream. Dehaene et al. (2005) proposed the “local combination detector” (LCD) model in which multiple levels of information of a word (e.g., fragments, features, single letters, bigrams, quadrigrams, and the whole word) are hierarchically encoded such that more anterior regions encode larger and more complex word components. The LCD model is supported by a functional magnetic resonance imaging (fMRI) study performed while subjects viewed alphabetic script; Vinckier et al. (2007) demonstrated that within the left occipitotemporal cortex, the posterior part is activated by false fonts, and the anterior part is activated by real words.

**Figure 1:**
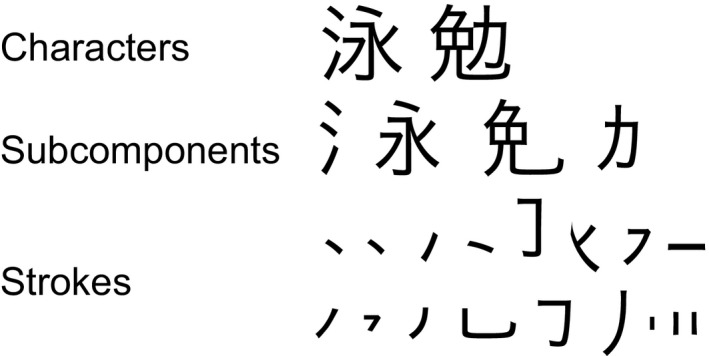
Examples of Japanese *Kanji* characters, subcomponents, and strokes. A Japanese *Kanji* character consists of subcomponents, which are combination of strokes.

It remains unclear, however, whether this model would be applicable to the case of a nonalphabetical language, for instance, Japanese *Kanji* characters. Logographic Japanese *Kanji* characters have dramatically different visual forms than alphabetic letters: they generally have a square shape and consist of subcomponents such as ‘radicals’ that are combinations of strokes. Japanese *Kanji* subcomponents are found in different *Kanji* characters (Fig. 1), and one or more subcomponents combine together to form a Japanese *Kanji* character. Additionally, the same subcomponents are used in many different *Kanji* characters. For example, one of the radicals “take‐kanmuri (i.e., 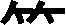)” is used in over 1000 *Kanji* characters. This leads to the possibility that the majority of *Kanji* characters are learned as a combination of *Kanji* subcomponents. Indeed, Tamaoka and Yamada (2000) reported that the extent of knowledge of *Kanji* radicals predicted spelling accuracy. Furthermore, *Kanji* subcomponents are the combinations of “strokes,” which are also visual components constructing *Kanji* characters. From the perspective of hierarchical visual processing system, *Kanji* characters are likely processed based on different levels of structural complexity. To our knowledge, hierarchical visual processing of Japanese *Kanji* characters has not been assessed with neuroscientific methods, but some researchers have investigated Chinese character processing with neuroimaging. Both Chinese and Japanese *Kanji* employ logographic strategies. The visual processing hierarchy of Chinese characters was investigated using real and pseudo Chinese characters and Korean Hangul. Real Chinese characters activated the anterior part of the fusiform gyrus, while Korean Hangul activated the posterior fusiform gyrus (Chan et al. 2009). Because Korean Hangul has a simpler visual structure than real Chinese characters, the authors compared the two writing systems to assess the hierarchical visual processing of Chinese characters. However, because they compared different languages, the findings might be due to differential structural components between the two languages. Another problem is that participants were required to complete different tasks for three types of stimuli (semantic or character form matching tasks). Therefore, the hierarchy demonstrated by the results might be dependent on the different cognitive strategies, such that semantic processing is only recruited for real characters. Liu et al. (2008) examined left occipitotemporal activation pattern for Chinese character using real, pseudo, and artificial Chinese characters. Although the activation peak in response to real Chinese characters was located in the anterior part of the left occipitotemporal cortex relative to the peak for pseudo Chinese characters, the peaks for pseudo and artificial characters were located in the same region. The methodological issue is that radicals were confounding their artificial Chinese character stimuli, resulting in the absence of contrast between the pseudo and artificial tasks. Thus, it has not been clearly demonstrated whether different visual components (e.g., stroke, *Kanji* subcomponents, and whole character) of logographic characters like *Kanji* are processed in different brain regions in a hierarchical manner.

Here, we used fMRI to examine whether *Kanji* characters are processed hierarchically from posterior to anterior in the left occipitotemporal cortex. Based on a study by Liu et al. (2008), we used a size judgment task to minimize semantic and phonological processing of *Kanji* information and only focused on its visual processing. In the size judgments task, participants were required to judge the size of the stimuli. This size judgment task does not require any intentional efforts to ‘read’ the characters, so the experiment implicitly relies on the automaticity of the reading process, which would likely have implications of the language network that would be activated for the different character types. A previous study which used such a size judgment task (Liu et al.^2008^) showed activation of language‐related brain regions, including the left inferior frontal gyrus and fusiform gyrus. Therefore, these brain regions can be considered as the network regarding automaticity of reading process. We exposed participants to (1) real *Kanji*, (2) pseudo *Kanji*, (3) artificial characters, and (4) a checkerboard pattern in the MRI scanner. We created pseudo *Kanji* and artificial characters by mixing components included in real *Kanji* characters. Pseudo *Kanji* characters are made by exchanging the subcomponents between two *Kanji* characters. Therefore, the characters contain radicals but do not exist as real *Kanji* characters. We further decomposed the subcomponents to create artificial characters that morphologically have the same contrast as a real *Kanji* characters (consisting of fragmented lines “strokes”) but do not have any structural components (i.e., *Kanji* subcomponentss) that are necessary to construct *Kanji*. Checkerboards consist of straight lines and only contain photic/optical components. If our hypothesis is correct, real *Kanji*, pseudo *Kanji*, and artificial characters should activate different parts of the left occipitotemporal cortex, and the activation peaks should be aligned in a sequential manner from posterior to anterior according to the components' complexity.

In addition, we investigated activations in different neuronal networks across the entire brain, centered on the left occipitotemporal cortex. For alphabetic script, real words produce functional connectivity among the VWFA, left superior temporal gyrus, and left inferior frontal gyrus (van der Mark et al. 2011). Another study used dynamic causal modeling (DCM) to show that Japanese real *Kanji* words induce effective connectivity across the left primary visual cortex, left ventral occipitotemporal cortex, and Broca's area (Duncan et al. 2013). A pseudo *Kanji* character has subcomponents information but does not exist as a real character. An artificial character has strokes but not subcomponents. It is reasonable to assume that these stimuli are processed in different neural networks (connections) than real *Kanji* characters. To our knowledge, no study has investigated differential neural networks for real *Kanji*, pseudo *Kanji*, and artificial character. We employed functional connectivity analyses to investigate whether these three types of stimulus facilitate connections in different neural networks.

## Materials and Methods

### Participants

Twenty‐eight right‐handed healthy native Japanese speakers (age *M *=* *21.7 years, *SD *= 2.3 years, age range: 18–28 years, 13 males) with normal or corrected vision and without language or speech impediments participated in the present study. Their handedness was affirmed with the Edinburgh handedness inventory (Oldfield 1971). We excluded 5 of the original 33 participants from the analyses because they failed to respond to the stimuli more than 5 times per session. All participants were undergraduate or graduate students who were already qualified by entrance exam to the universities, so that they are supposed to have sufficient sight word reading ability equally. This study was approved by Research and Ethical Committee of University of Tsukuba and National Center of Neurology and Psychiatry, and written informed consent was obtained from each participant prior to the study.

### Stimuli

**Figure 2:**
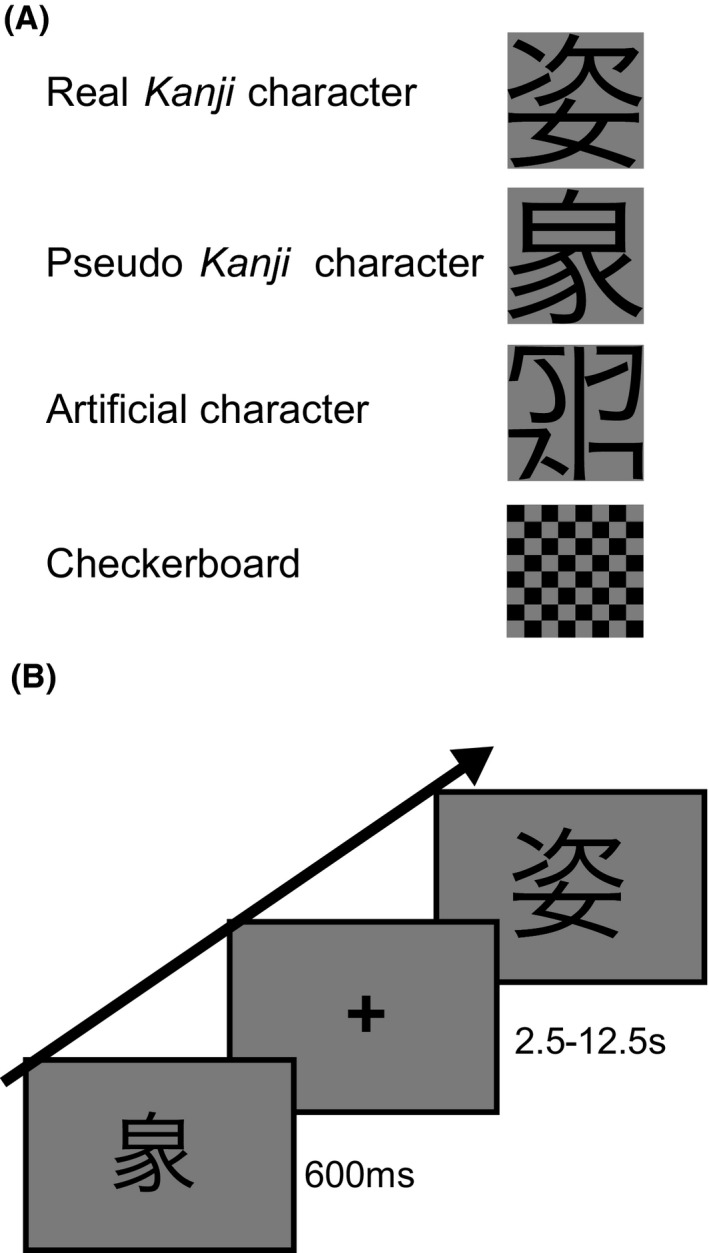
Stimuli examples and schematic illustration of the experimental design. (A) This figure shows four types of presented stimuli. We exposed real *Kanji* characters (complete structure), pseudo *Kanji* characters (random combinations of subcomponents), artificial characters (character fragments), and a checkerboard (simple photic stimuli). (B) During the fMRI session, participants were required to judge if the presented stimuli were large or small.

We created four types of stimulus: (1) real *Kanji*, (2) pseudo *Kanji*, (3) artificial character, and (4) a checkerboard pattern (Fig. 2A). Eighty *Kanji* characters with 8–12 strokes were chosen for the real *Kanji* character stimuli. In this study, a psycholinguistic database of lexical properties of Japanese (Amano and Kondo 1999, 2000) was used to examine the familiarity and frequency of these *Kanji* characters. This database includes subjective rating data for word familiarity, character familiarity, and visual complexity. For objective scale, this database contains word and character frequency in the newspaper. We collected highly familiar and frequent characters (>1 SD from the mean in the database) because these characters are used frequently in daily life. Additionally, all *Kanji* characters were chosen from 2136 commonly used *Kanji* which Japanese students commonly learn to read and write. This may attenuate startle effect of the stimuli. Both pseudo *Kanji* and artificial characters were created by recomposing subcomponents of real *Kanji* characters. Pseudo *Kanji* characters were made by replacing the subcomponents of a real *Kanji* character with a radical of another real *Kanji*, such that the pseudo *Kanji* stimuli include real subcomponents but are not fully structured as real *Kanji* characters. We prepared artificial characters by decomposing *Kanji* subcomponents s first and then randomly recomposing them. Hence, artificial characters were unpronounceable, meaningless, and never included *Kanji* subcomponents. Thus, unlike the study by Liu et al. (2008), we decomposed artificial characters into a completely non‐linguistic level, which enabled us to evaluate whether *Kanji* characters and subcomponents would activate different brain regions within the left occipitotemporal cortex. Half of the stimuli were 20% larger in terms of size than the other half, so we obtained even sets of “large” and “small” characters.

### Experimental design

The participants were put in the scanner and then experienced four consecutive fMRI sessions, each of which consisted of the visual presentation of 20 real *Kanji* characters, 20 pseudo *Kanji* characters, 20 artificial characters, and 20 checkerboard stimuli. Each stimulus was presented for 600 ms, followed by the presentation of a fixation cross (Fig. 2B). The time periods of the presentation of fixation cross were randomized based on TR ranging from 2.5 to 12.5 s. The orders of the four types of stimuli were randomized across sessions. Participants were required to judge the size of the character (large or small) and press a response button with his/her right index or middle finger if the stimulus was large or small, respectively. During the fixation periods, participants maintained their gaze on the fixation point without any response. Sessions in which the participant did not respond over five times were excluded from later behavioral and fMRI data analyses. To confirm if cognitive efforts for this size‐judgment task were equal between different character types and did not influence hierarchical *Kanji* processing, the reaction time and error rate were analyzed using two‐way factorial analysis of variance (ANOVA) of character type (real, pseudo, anartificial, checkerboard) × size (large and small). Statistical threshold for behavioral data was set at *P *<* *0.01. We used SPSS ver. 22 (IBM Corp., Armonk, NY, USA) for all statistical analyses.

### fMRI data acquisition and analysis

#### fMRI acquisition

We obtained functional echo planar images (EPI) using a Siemens (Erlangen, Germany) 1.5‐Tesla MRI system (TR = 2500 ms, TE = 40 ms, flip angle 90°, 4‐mm‐thick axial slices, in‐plane resolution = 3.4 × 3.4 mm). The time‐series of functional data of 190 whole‐brain 3D EPI volumes were acquired in each session. After functional image acquisition, a T1‐weighted high‐resolution anatomical image was obtained (TR = 1900 ms, TE = 3.93 ms, flip angle 15°, voxel size = 1.4 × 1.0 × 1.3 mm).

#### fMRI data analysis

We analyzed the functional imaging data using SPM8 (Wellcome Department of Cognitive Neurology, London, UK, http://fil.ion.ucl.ac.uk/spm/). The first five images were excluded from further analyses to avoid T1 equilibration effects. To compensate the differential temporal offsets between slices, we applied slice‐timing correction to the images. Then we spatially realigned the functional images to the first image for motion correction and coregistered the functional images to the individual structural image (high‐resolution T1). The individual structural image was spatially normalized to the MNI T1 template image. By using the resulting normalization parameters, all functional images were spatially normalized. Finally, functional images were smoothed using a Gaussian filter (6‐mm FWHM).

To model the hemodynamic time series in each condition, we convolved the stimulus presentation time (600 ms) with the canonical hemodynamic response function (HRF). Individual hemodynamic changes for each of four types of stimulus events (real *Kanji*, pseudo *Kanji*, and artificial characters and checkerboard) were assessed using a session‐wise general linear model that included the linear combination of the regressors for the hypothetical hemodynamic responses to the four types of events, motion parameters, and high‐pass filtering regressors (128 s). We estimated the parameters (beta‐weights) for the four conditions, and the parameters were fed into the group analysis.

First we tried identifying the commonly activated regions across the three types of different stimulus events (real *Kanji*, pseudo *Kanji*, and artificial characters) relative to checkerboard. All beta‐weight maps for the three conditions in comparison to checkerboard were fed into a conjunction analysis. The height threshold was set at *P *<* *0.005 (uncorrected), and the cluster size threshold was *P *<* *0.05 corrected with family wise error (FWE) (536 or more voxels). Finally, we investigated the differential loci of the peak activation for the three stimulus conditions to see if there was a topologically hierarchical peak distribution. The beta‐weight maps for the three stimulus conditions were compared using one‐way repeated measures ANOVA. For this analysis, the height statistical threshold was set at *P *<* *0.001, uncorrected, and the cluster size threshold was set at *P *<* *0.05 with FWE corrected (463 or more voxels).

#### Region of interest analysis

To differentiate spatial patterns of activation for real *Kanji*, pseudo *Kanji*, and artificial characters, five non‐overlapping regions of interest (ROIs) were defined based on a previous study (Van der Mark et al. 2009). These ROIs cover the putative VWFA and the neighboring region. Each spherical ROI had a 3‐mm radius, and all the ROIs were located along with the posterior–anterior axis (slightly declined toward the anterior direction). The center coordinates for the ROIs are as follows: (MNI coordinate [*x*,* y*,* z*] in mm) ROI1 (−45, −75, −12), ROI2 (−45, −67, −13), ROI3 (−45, −59, −14), ROI4 (−45, −51, −15), and ROI5 (−45, −43, −16). ROI3 was located on the proper VWFA region for Japanese *Kanji* characters that was described in a previous meta‐analysis of culturally specific word regions (Bolger et al. 2005). Similarly, contralateral coordinate were defined as the center coordinate for ROIs of the right occipitotemporal cortex. The ROI definition and parameter estimates were conducted using the MarsBaR toolbox (http://marsbar.sourceforge.net).

#### Functional connectivity analysis

To identify the neural networks engaged in different components of visual *Kanji* processing, we conducted functional connectivity analyses (i.e., the correlations of time courses in the seed ROIs with those in voxels across the entire brain). To compare functional connectivity between real *Kanji*, pseudo *Kanji*, and artificial characters, seed‐based correlation analyses were performed using functional connectivity toolbox version 12 (http://web.mit.edu/swg/software.htm). Because real *Kanji*, pseudo *Kanji*, and artificial characters had been expected to induce peaks in different regions, we created seed regions for functional connectivity analyses after we obtained the peaks of event‐related activations. The seed for real *Kanji* characters was defined as a 3‐mm‐diameter sphere centered on the peak coordinate shown in real *Kanji* character minus checkerboard contrast. The seeds for the pseudo *Kanji* and artificial characters were generated in the same manner, using pseudo *Kanji* character versus checkerboard and artificial character versus checkerboard contrast, respectively. After data preprocessing, the data were band‐pass filtered between 0.008 and 0.2 Hz, which was the effective frequency range of task‐related hemodynamic change as examined with a spectral analysis. Functional connectivity was estimated by a regression analysis co‐varied with the main event‐related task effect (i.e., the modeled hemodynamic response to the judgment events) and other nuisance variables (including white matter and cerebrospinal fluid signals and six head motion parameters). Thus, the resulting maps represent voxels within the brain in which the hemodynamic response during the fMRI sessions correlated with that of the seed regions. The height threshold was *P *<* *0.0005 (uncorrected), and the extent threshold was *P *<* *0.05 (corrected, *k *>* *29) based on the results of 5000 iterations of Monte Carlo simulation performed on ANFI's AlphaSim (http://afni.nimh.nih.gov/pub/dist/doc/manual/AlphaSim.pdf), with single voxel *P *<* *0.0005, FWFM = 6 mm, cluster correction radius in 4 mm, and brain mask.

## Results

### Behavioral data of the size judgment task

Accuracy of all conditions was almost 100% (Table 1). Indeed, size judgment task accuracy did not differ significantly between different character sizes (large or small) or types (real, pseudo, artificial, or checkerboard), *F*(1, 27) = 1.34, *P *=* *0.257 and *F*(1.4, 38.2) = 5.04, *P *=* *0.020, respectively. We found interactive effect of size × types, *F*(1.62, 27) = 8.94, *P *=* *0.001, such that, although accuracy in response to large and small stimuli is mostly identical to each other on three types of characters, accuracy to large stimuli (98.3%) was a little lower than that to small stimuli (99.6%) on the checkerboard condition. We cannot put the plausible explanation for this lower accuracy judging larger checkerboard, but there were no interactive effect of size × type at least within three character types on accuracy [*F*(2,54) = 0.216, *P *=* *0.806].

**Table 1:** Mean (SE) of response accuracy and response time in each stimulus type/size

	Real	Pseudo	Artificial	Checkerboard
	M (SE)	M (SE)	M (SE)	M (SE)
Accuracy (%)				
Big	99.6 (0.1)	99.4 (0.1)	99.5 (0.1)	98.3 (0.4)
Small	99.5 (0.2)	99.3 (0.2)	99.4 (0.2)	99.6 (0.1)
RT(ms)				
Big	635.8 (20.2)	644.9 (21.6)	638.5 (20.1)	680.8 (28.5)
Small	668.6 (20.4)	676.5 (21.7)	683.6 (20.0)	651.1 (19.0)

M, mean; SE, standard error.

The reaction time did not differ between the character sizes (large or small) and types (real, pseudo, artificial, or checkerboard), *F*(1, 27) = 4.25, *P *=* *0.049 and *F*(3, 81) = 1.31, *P *=* *0.278, respectively. There was an interactive effect of size × type on the reaction time, however, *F*(1.8, 49.6) = 7.11, *P *=* *0.002, such that participants responded faster to larger character stimuli than smaller characters although they responded slower to larger checkerboard stimuli than to smaller checkerboards (Table 1). As in the case with the interactive effect on accuracy, we could not find any interpretable reason for this interaction on reaction time. But there were no interactive effect of size × type within three character types on reaction time [*F*(2,54) = 0.417, *P *=* *0.661].

Therefore, participants performed equally in terms of response accuracy and speed, at least for the three character types of stimuli that are focus of our study. Because size judgement is not a scope of this study, both big and small size conditions were fed into the same conditions in the fMRI analysis.

### fMRI data

#### Neural activations common to all types of Kanji characters

**Figure 3:**
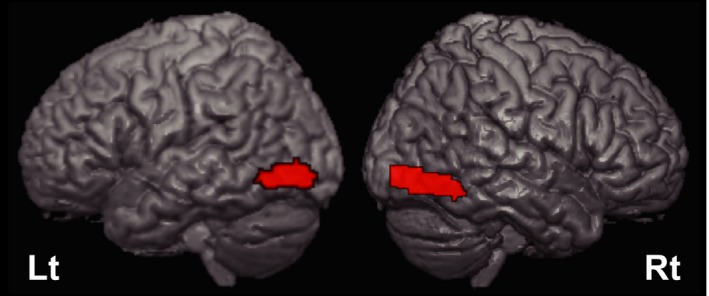
Common brain activation maps among the real *Kanji*, pseudo *Kanji*, and artificial characters in comparison to the checkerboard. Bilateral occipitotemporal cortices were commonly activated across the real *Kanji*, pseudo *Kanji*, and artificial characters in comparison to the checkerboard. The height threshold for illustration was *P *<* *0.005, (uncorrected), with a cluster‐size threshold of *P *<* *0.05 (corrected) with family wise error (FWE, 536 or more voxels).

To identify brain regions engaged in processing the three different types of *Kanji* characters, we contrasted three activation maps (for real *Kanji*, pseudo Kanji, and artificial characters) with the checkerboard map, and computed common activation areas across the three contrasts (Fig. 3 and Table 2). We were able to confirm that the task we created activated the bilateral occipital gyrus and fusiform gyrus.

**Table 2:** Common brain activation in response to the real *Kanji*, pseudo *Kanji*, and artificial characters in comparison to the checkerboard

Region	MNI coordinates (mm)			*T*	Voxels	BA
	*x*	*y*	*z*			
R inferior temporal gyrus	42	−66	−10	6.56**	845	37
R inferior occipital gyrus	40	−82	−8	5.86**		19
R fusiform gyrus	48	−52	−14	4.50*		37
L inferior occipital gyrus	−40	−78	−6	4.72*	536	19
L fusiform gyrus	−42	−54	−10	3.76*		37
L sub‐gyral	−42	−44	−12	3.70*		37

Co‐activated brain regions across real *Kanji*, pseudo *Kanji*, and artificial characters in comparison to checkerboard. Bilateral occipitotemporal cortices were commonly activated.

*P <* *0.005, *k *>* *20, **P <* *0.05 (FWE corrected).

#### Different spatial patterns of activation within the left occipitotemporal cortex for all Kanji character types

**Figure 4:**
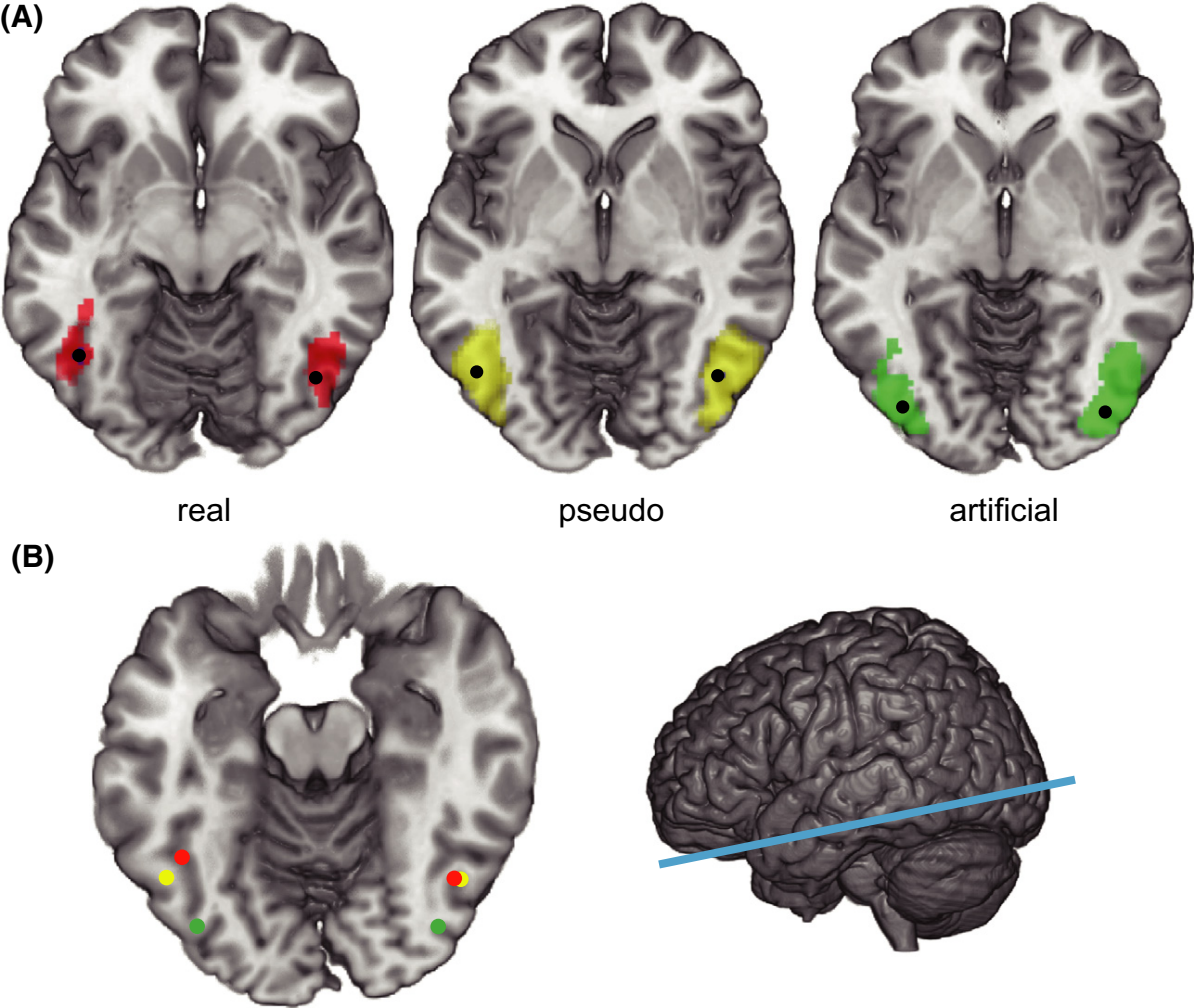
Activation clusters and the peaks for the real *Kanji*, pseudo *Kanji*, and artificial characters relative to the checkerboard. (A) The figures show the activated regions in response to the real *Kanji*, pseudo *Kanji*, and artificial characters contrasted with the checkerboard. Black dots indicate the peaks of the activated clusters. The height threshold was set at *P *<* *0.001 (uncorrected), and the cluster size threshold was set at *P *<* *0.05 (corrected with FWE, 463 or more voxels). Real, real *Kanji* character; Pseudo, pseudo *Kanji* character; Artificial, artificial character. (B) Overlay of the activation peaks on an axial slice (blue line in the right panel). The peaks for the real, pseudo, and artificial characters are colored in red, yellow, and green, respectively.

**Figure 5:**
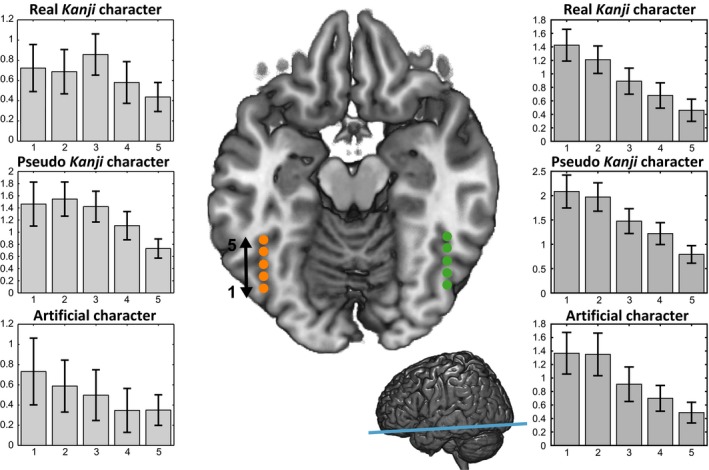
ROI analysis in the bilateral occipitotemporal cortices. The consecutive numbers 1–5 on the brain slice section indicate the ROI number, such that the most posterior ROI is numbered 1 (ROI1) and the most anterior ROI is numbered 5 (ROI5) in the occipitotemporal cortex along the posterior–anterior axis (blue line on the right figure). The three bar graphs show the contrast estimates of the neural activity in ROIs 1–5 in response to real *Kanji*, pseudo *Kanji*, and artificial characters. The error bars represent standard errors across participants.

For a more detailed illustration of the spatial distribution of activation for real *Kanji*, pseudo *Kanji*, and artificial characters in the bilateral occipitotemporal cortices, we mapped peaks of the brain activity for each stimulus relative to checkerboard (Fig. 4 and Table 3). The activation clusters for real *Kanji*, pseudo *Kanji*, and artificial characters were overlapped (Fig. 4A). However, once they were overlaid on an axial slice, the peak regions for the artificial, pseudo *Kanji*, and real *Kanji* characters appeared to be located in the left occipitotemporal cortex in a posterior to anterior order (Fig. 4B). Conversely, the peak locations for real and pseudo *Kanji* characters in the right occipitotemporal cortex were adjacent to each other, suggesting that hierarchical localization of the activations was dominant in the left side of the occipitotemporal cortex. To statistically assess whether the three types of stimuli produced different spatial patterns of brain activity only in the left inferior occipital temporal cortex, we created consecutive ROIs within the region and compared the spatial patterns of activation across the three conditions (Fig. 5). The results clearly showed that the three types of characters produced significantly different spatial activation patterns. Two‐way repeated ANOVA of the parameter estimate of activations revealed an interactive effect of ROI locations (1–5) × character types (real, pseudo, and artificial), *F*(8,216) = 3.847, *P *=* *0.009. This interaction was explained by the differential spatial distributions (i.e., different locations for the most activated ROI) across the three types of characters (Fig. 5, left). For artificial characters, the most activated ROI was in the extreme posterior region (ROI1), and brain activities were decreased along the posterior‐to‐anterior axis. Pseudo *Kanji* characters evoked the strongest activity in the second ROI from the posterior region (ROI2). Conversely, real *Kanji* characters elicited the strongest activity in the middle ROI (ROI3), which corresponds to putative center of the VWFA for Japanese *Kanji* characters. In the right occipitotemporal cortex, however, we did not find interactive effect of ROI locations (1–5) x character types (real, pseudo, and artificial). As we expected, the result strongly supports the hypothesis that artificial, pseudo *Kanji*, and real *Kanji* characters are processed hierarchically from posterior to anterior in the left occipitotemporal cortex.

**Table 3:** Brain regions activated by each stimulus type

Region	MNI coordinates (mm)			*T*	Voxels	BA
	*x*	*y*	*z*			
Real > Checkerboard						
R inferior temporal gyrus	42	−66	−10	6.56*	713	37
R inferior occipital gyrus	40	−82	−8	5.86*		19
R fusiform gyrus	48	−52	−14	4.50*		37
L fusiform gyrus	−44	−58	−12	4.83*	463	37
L inferior occipital gyrus	−40	−78	−6	4.72*		19
L sub‐gyral	−42	−40	−10	4.01*		37
Pseudo > Checkerboard						
R inferior temporal gyrus	44	−66	−10	9.70**	1353	37
R inferior occipital gyrus	42	−78	−8	8.02**		19
R fusiform gyrus	46	−56	−12	7.68**		37
L inferior temporal gyrus	−48	−64	−10	8.89**	1342	37
L inferior occipital gyrus	−40	−80	−6	6.54**		19
L fusiform gyrus	−36	−42	−22	4.48*		20
Artificial > Checkerboard						
R inferior occipital gyrus	38	−82	−8	7.63*	1033	19
R middle temporal gyrus	42	−64	−8	7.23*		37
R fusiform gyrus	46	−54	−12	5.10*		37
L inferior occipital gyrus	−40	−80	−6	6.55*	522	19
L fusiform gyrus	−40	−44	−12	3.89*		37
L fusiform gyrus	−42	−54	−10	3.76*		37

*P <* *0.001 (uncorrected), cluster size threshold of *P *<* *0.05 (FWE corrected) **P <* *0.05 (FWE corrected) for the height threshold.

#### Functional connectivity analyses seeded in the left occipitotemporal cortex

**Figure 6:**
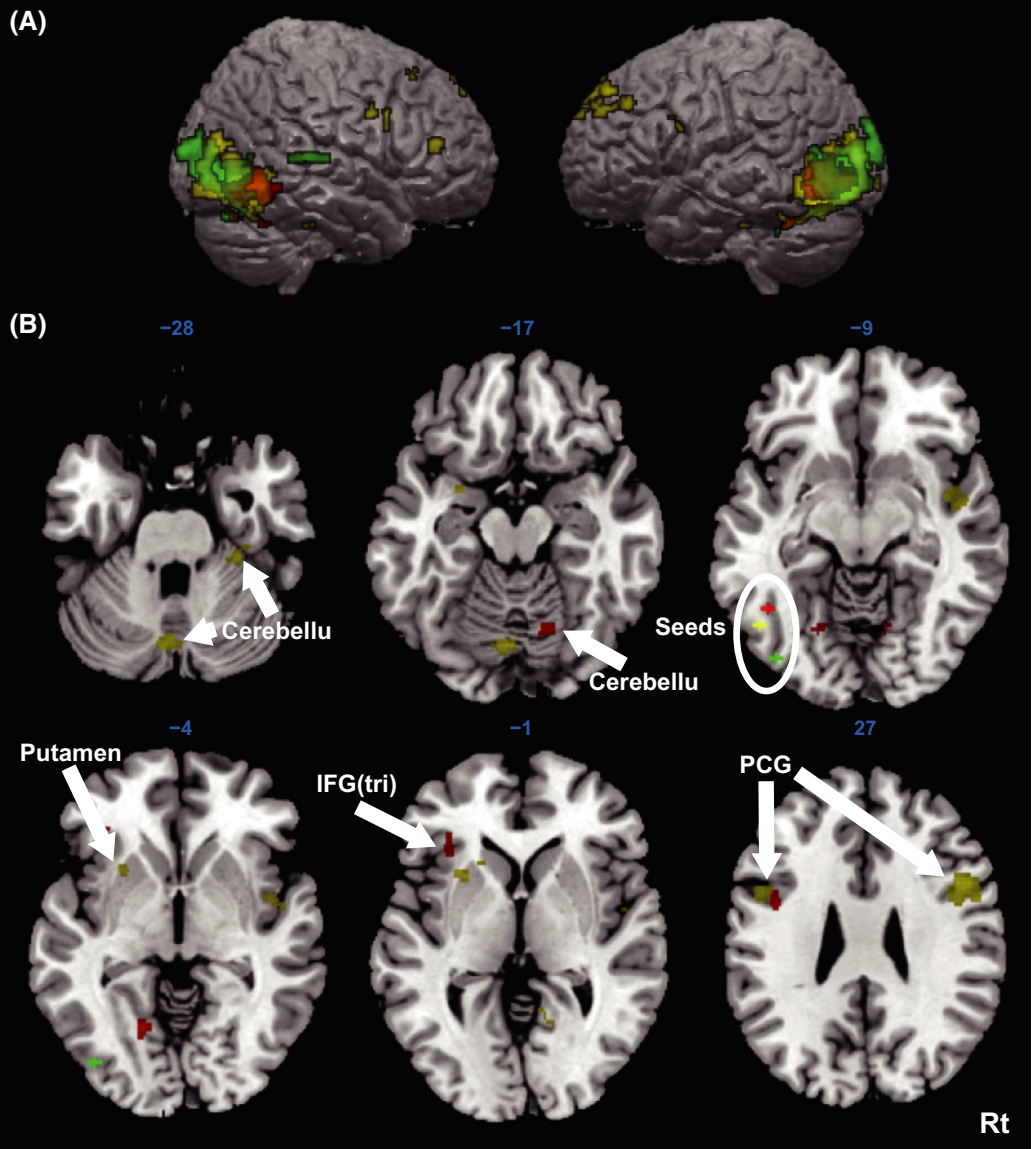
Functional connectivity analyses seeded in the left occipitotemporal cortex. The colored blobs on the brain maps show the areas with significant functional connectivity to the seed regions in the left occipitotemporal cortex. The red blobs show the areas with differential functional connectivity between the real *Kanji* and the checkerboard. Yellow indicates areas with differential functional connectivity between pseudo *Kanji* and checkerboard, and the green indicates differential functional connectivity between artificial characters and checkerboard. The height threshold was *P *<* *0.0005 (uncorrected) and the extent threshold was *P *<* *0.05 (corrected; *k *>* *29, AFNI AlphaSim). (A) Lateral view. The auto‐ and contralaterally correlated connections were found within the bilateral occipital gyri, inferior temporal gyri, and fusiform gyri. (B) Remote brain regions connected to the left temporal seeds. The auto‐ and contralaterally correlated brain regions were omitted for illustrative purposes. IFG (tri), inferior frontal gyrus (triangular); PCG; precentral gyrus.

To investigate which brain regions are remotely related to the hierarchical processes, we separately computed functional connectivity for real *Kanji*, pseudo *Kanji*, and artificial characters. The seed regions were set at the peak locations for the three character types in the left occipitotemporal cortex (Fig. 4B). First, we confirmed that the activities around each seed were strongly autocorrelated with the activities of the seed itself and the contralateral corresponding region within the bilateral occipital gyri, inferior temporal gyri, and fusiform gyri (Fig. 6A). We found that the three seeds were functionally connected to remote regions differently (Table 4): the seed for real *Kanji* characters was connected to the left inferior frontal gyrus (triangularis), which was not connected to the seeds for the pseudo characters or artificial *Kanji* characters (Fig. 6B). The seed for pseudo *Kanji* characters was functionally connected to multiple brain regions, including the bilateral cerebellum, bilateral inferior frontal gyri, left putamen, left superior temporal gyrus, and right middle frontal gyrus. The artificial character seed was only connected to the right superior temporal gyrus. As we predicted, the seeds for the real *Kanji* characters in the left inferior temporal region were connected to remote language‐related brain regions, although pseudo *Kanji* characters induced different connections to remote regions, including the basal ganglia and cerebellum.

**Table 4:** Regions with functional connectivity to the inferior temporal region during observation of the three character types

Region	MNI coordinates (mm)			*T*	Voxels	BA
	*x*	*y*	*z*			
Real *Kanji* character						
R cerebellum	16	−66	−18	4.52	47	
L lingual gyrus	−16	−62	−4	5.17	41	19
L inferior frontal gyrus (triangular)	−34	30	2	4.66	35	47
L precentral gyrus	−44	2	32	5.69	66	9
Pseudo *Kanji* character						
L cerebellum	−6	−72	−22	6.96	253	
R cerebellum	30	−32	−28	4.59	61	
L amygdala	−26	0	−16	4.61	31	
R superior temporal gyrus	48	−6	−8	4.57	78	22
L putamen	−28	12	−2	5.08	63	
R calcarine fissure	22	−62	10	5.94	179	30
R inferior frontal gyrus (triangular)	50	40	16	4.50	32	8
R precentral gyrus	48	10	32	7.15	226	9
L precentral gyrus	−50	4	26	5.14	79	9
L superior occipital gyrus	−18	−76	32	5.04	64	7
L superior frontal gyrus	−22	46	44	5.50	268	8
R middle frontal gyrus	34	26	54	4.79	32	8
Artificial character						
R superior temporal gyrus	68	−26	8	4.53	41	42

The height threshold was *P *<* *0.0005 (uncorrected) and the extent threshold was *P *<* *0.05 (corrected; *k *>* *29, AFNI AlphaSim). The auto‐ and contralaterally‐correlated brain regions were omitted.

## Discussion

The purpose of the present study was to examine whether the visual forms of Japanese *Kanji* are processed hierarchically. We found that, in the left occipitotemporal coortex, the peak regions for the artificial, pseudo *Kanji*, and real *Kanji* characters were localized in a posterior to anterior order, although this hierarchical organization was not found in the right occipitotemporal cortex. The ROI analysis of the left occipitotemporal cortex statistically confirmed our hypothesis such that the spatial activation patterns (i.e., the patterns of activations across five consecutive ROIs) were different between the stimulus types and the most activated ROIs for the three stimulus types lined up in order.

Some studies have shown hierarchical activation for visual word forms in the left occipitotemporal cortex for alphabetic script (Vinckier et al. 2007). For instance, Thesen et al. (2012) showed real words, pseudo words, and false fonts to participants in the scanner and observed hierarchical formation of the left fusiform gyrus. Pseudo words are meaningless but contain real letters. However, false fonts contain letter‐like symbols, so the authors reasoned that letterforms would be indexed by the contrast of pseudo word versus false fonts, whereas word forms would be indexed by the contrast of real words versus pseudo words. They performed magnetoencephalography (MEG) and observed a letter‐selective response occurring 60 ms earlier than the word‐selective response, strongly suggesting temporally sequential processing of “letter” and “word” information in the left occipitotemporal cortex. Although we found interaction between ROIs and stimulus types, interpretation of this interaction remains only descriptive. Together with our peak analysis and previous studies, however, it is reasonable to think that results indicate hierarchical organization of Japanese *Kanji* character. Moreover, the peak for the real *Kanji* characters (*x *= −44, *y *= −58, *z *= −12) in our study was very close to the word form area reported by Thesen et al. (*x *= −46, *y *= −52, *z *= −20) (~10 mm apart). Similarly, the peak for the pseudo *Kanji* characters (*x *= −48, *y *= −64, *z *= −10) was not far from the letter form area (*x *= −40, *y *= −78, *z *= −18) with a distance of ~16 mm. Based on our findings, we suppose that, in terms of character form, an alphabetic word corresponds to an entire *Kanji* character in Japanese, and an alphabetic letter corresponds to *Kanji* subcomponents.

Because of the visual complexity of Japanese *Kanji* characters, one study suggested that they induce right lateralized activation (Nakamura et al. 2005). In the present study, however, we observed different peaks for different character types in the left occipitotemporal cortex, which was also confirmed by the ROI analysis. Evidence from brain lesion studies supports our hypothesis of left dominance for visual processing, even for Japanese *Kanji*; lesions in the left inferior temporal area cause alexia with agraphia for Japanese *Kanji* characters (Iwata 1984), and patients with damage to the left inferior temporal region exhibit poor lexical decision performance (Tani 2004). In addition, Korean cerebrovascular disease patients with damage to the left inferior temporal region show decreased performance when reading logographic *Hanja* (Korean *Kanji*) (Kwon et al. 2002). Collectively, the existing evidence indicates that visual forms of Japanese *Kanji* characters may be processed in the same manner in the left hemisphere as alphabetic scripts.

Another aspect to be concerned is the semantic and phonological information which each Japanese *Kanji* character has. Recent neuroimaging studies have shown that the fusiform gyrus is involved in phonological processing. The Japanese *Kanji* characters we used in our study can be read aloud, while pseudo *Kanji* and artificial characters do not contain phonological information at the whole character level. The different activation peak loci for the three types of *Kanji* character might be due to differences in phonological processing induced by the three types of characters. However, a neuroimaging study showed that brain activity in the left occipitotemporal cortex was elicited by a reading pseudoword but not reading words and silent reading (Dietz et al. 2005). Reading pseudowords requires grapheme to phoneme conversion, although reading real word can be achieved by using lexical knowledge. This implies that activation related to phonological processing in the fusiform gyrus is supposed to be task dependent. Similarly, semantic information might induce different activation peak loci, because real *Kanji* stimuli we used were highly familiar ones which perhaps easily activate semantic networks. In the present study, we exposed each stimulus for limited time period (600 ms) and we required the participants to judge the size of the stimulus in order to limit the participants' time to read the stimulus with their “internal voice” or to recruit semantics of the stimuli. Nevertheless, stimulus presentation time may affect the cognitive process, that is, the different phonological and or semantic information between the character types could have affected activity in the left occipitotemporal cortex to some extent. More generally, however, existence of semantic and phonological information in each Japanese *Kanji* characters is a characteristic of this language system and we cannot completely distinguish between visual information of *Kanji* forms and semantics and/or phonological information.

Although we found different peak loci in the left occipitotemporal region, pseudo *Kanji* character produced stronger activation than real *Kanji* and artificial character. One of the conceivable possibilities of this strong activation is the visual novelty of the stimuli. Some researchers reported that object novelty activates the fusiform gyrus more than familiar objects (Zhang et al. 2013). Considering that pseudo characters created in this study do not exist in reality, these characters are supposed to be more novel than real *Kanji* characters. Thus, novelty of visual stimuli may induce larger brain activity for pseudo *Kanji* character. Moreover, because a pseudo *Kanji* character consists of multiple pronounceable subcomponents of real *Kanji* characters, we could speculate that it might activate larger phonological process in occipitotemporal cortex. Further studies are needed to clarify such speculative ideas, such as the effect of stimulus novelty and subjects' implicit attempts to pronounce pseudo *Kanji* character with multiple pronounceable subcomponents on visual word processing.

We found that activations in the left occipitotemporal cortex were differentially connected to remote brain regions, depending on the types of characters (real *Kanji*, pseudo *Kanji*, or artificial characters). We observed functional connectivity between the left inferior temporal region and left inferior frontal language area for the real *Kanji* characters (but not for the pseudo *Kanji* or artificial characters). This result is consistent with previous studies in which functional connectivity was observed between the left fusiform gyrus and left inferior frontal gyrus when the participants performed rhyme judgments and a lexical decision task (Booth et al. 2007; Duncan et al. 2013). A diffusion tensor imaging study showed anatomical connectivity between the primary visual cortex and inferior temporal gyrus via the middle and inferior temporal gyri (Yeatman et al. 2013), which is also consistent with our results. The left inferior frontal gyrus is thought to be involved in phonological processing (Tan et al. 2001; Gold and Buckner 2002; Turkeltaub et al. 2003; Matsuo et al. 2010). In our study, only the real *Kanji* characters possessed the complete set of visual components, including phonological information. Therefore, such information may automatically initiate functional connectivity with remote language areas, such as the left inferior frontal gyrus. We did not find any explicit local activity related to phonological or motor speech function (because phonological processes were suppressed in this study), but the brain might be, at least, “ready” to access and output the phonological and/or semantic information of the real *Kanji* characters by initiating connectivity between remote regions. Accordingly, phonological information in visual word recognition reaches a speech‐related remote region (pars opercularis of the inferior frontal gyrus) very promptly (~100 ms), which has been shown by a MEG study (Wheat et al. 2010).

We found that the right cerebellum was functionally connected to the left inferior temporal seed region. Although the cerebellum has been regarded as being involved in motor control (Stein 1986), recent neuroimaging studies have revealed its involvement in non‐motor functions such as language, learning, and memory (Desmond and Fiez 1998; Frings et al. 2006). Bilateral cerebellar activation during a rhyme judgment task suggests that the cerebellum is engaged in phonological learning (Fulbright et al. 1999). In this regard, the cerebellum may work together with the inferior frontal regions for phonological processing. Anatomical investigations using transneuronally transported viruses in monkeys (Kelly and Strick 2003) and diffusion tensor imaging studies (Doron et al. 2010) have demonstrated a neuronal projection from the cerebellum to the anterior frontal lobe. A functional neuroimaging study also showed reciprocal connectivity between the cerebellum and left inferior frontal gyrus during a rhyme judgment task (Booth et al. 2007). Together with our results, the evidence indicates that left inferior temporal regions may cooperate with the inferior frontal gyrus and right cerebellum for the phonological processing of real *Kanji* characters.

In contrast, the pseudo *Kanji* characters produced functional connections with distinct brain regions, such as the bilateral cerebella, left putamen, bilateral precentral gyri, and bilateral middle frontal gyri. It is well‐known that brain activity in visual regions is modulated by a task that invokes top‐down control from the prefrontal cortex (Desimone and Duncan 1995; Morishima et al. 2009). We found a functional connectivity between the left inferior temporal seed and distributed frontal areas, including the right middle frontal gyrus corresponding to Brodmann areas 8 and 9. Gazzaley et al. (2007) found that functional connectivity between prefrontal and visual areas during encoding faces or scenes was stronger than connectivity that occurred while passively viewing the stimuli, suggesting that prefrontal‐visual connectivity may be involved in learning visual stimuli. The pseudo *Kanji* characters we used were legitimate at the radical level, but they do not exist at the character level and would be novel visual stimuli. Therefore, the functional connectivity to Brodmann areas 8 and 9 might reflect the automatic processes that facilitate learning a novel visual stimulus as a new *Kanji*.

We also found that pseudo *Kanji* characters initiated functional connectivity from the left inferior temporal to the left putamen. The basal ganglia, including the putamen, constitutes a critical component of the cortico‐basal ganglia‐thalamus (CBT) loop (Haber and Calzavara 2009). Although this loop was originally considered to engage in motor control (DeLong et al. 1983), it is now believed to be involved in more advanced features such as planning, language learning, and reward (Rolls 2000; Tekin and Cummings 2002; Grace et al. 2007). The basal ganglia act as a “gating switch” to regulate even non‐motor functional connections between remote regions across cortices (Grace 2000), which was confirmed by a computational modeling study (O'Reilly and Frank 2006). van Schouwenburg et al. (2010) demonstrated that basal ganglia activity modulates top‐down signals from prefrontal to visual areas. A novel stimulus like a pseudo *Kanji*, which might initiate some learning processes as discussed above, is supposed to engage the functional connectivity to the putamen that might mediate and/or regulate top‐down signals from the frontal lobe to the visual cortex.

Another functional connectivity we observed for pseudo *Kanji* characters was between the inferior temporal seed and the cerebellum. As mentioned before, the cerebellum is thought to be involved in phonological processing. Exposure to auditory novel pseudo words increased cerebellar activation, suggesting that the cerebellum is involved in phonological learning (Rauschecker et al. 2008). Another study also demonstrated fusiform‐cerebellum connectivity during a rhyme judgment task (Booth et al. 2007). The cerebellum is reciprocally connected with the lateral inferior temporal region and the inferior frontal gyrus, so phonological information is likely to be processed in this cerebellar‐inferior frontal loop. The cerebellar loop is involved in motor learning and other types of learning (Doya 2000). As discussed above, a pseudo *Kanji* character is visually similar to a real *Kanji* character, and the participants may recognize the pseudo character as a “new character.” This would implicitly introduce the visual information to the phonological learning process, which enhances functional connectivity from the inferior temporal lobe to the cerebellum. In sum, enhanced functional connectivity in response to pseudo *Kanji* characters is supposed to reflect the automatic phonological and visual learning processes. On the other hand, real *Kanji* characters do not initiate the learning process (because they are already known); rather, they connect the visual regions to the regions related to more advanced processes to output the information, such as language regions. These ideas here remain speculative and warrant further study in the future.

## Conclusion

We found different peak locations for real *Kanji*, pseudo *Kanji*, and artificial characters spatially aligned in order of the structural organization of *Kanji* characters within the left occipitotemporal cortex, suggesting hierarchical visual processing of Japanese *Kanji* characters. Moreover, the three stimulus types facilitate different neuronal networks or functional connectivity. Future studies will be expected to examine the different components constructing *Kanji,* taking into account the effects of phonological, semantic, and learning processes and stimulus familiarity on left occipitotemporal cortex activities.

## Conflict of Interest

None declared.

## Supporting information


**Figure S1.** Functional connectivity analyses seeded commonly activated brain region in the left occipitotemporal cortex.
**Table S1.** Regions with functional connectivity from the inferior temporal region during observation of the three character types.
