# Supplementary material for: Neural basis of hierarchical visual form processing of Japanese Kanji characters
Source: Brain Behav. 2015 Nov 4;5(12):e00413. doi: 10.1002/brb3.413 (PMC4714641; doi:10.1002/brb3.413)
Supplement: Supplementary file 1 — Figure S1. Functional connectivity analyses seeded commonly activated brain region in the left occipitotemporal cortex. Table S1. Regions with functional connectivity from the inferior temporal region during observation of the three character types. [file BRB3-5-e00413-s001.doc]

**Supplemental Data**

**Neural basis of hierarchical visual form processing of Japanese *Kanji* characters**

Hiroki Higuchi, Yoshiya Moriguchi, Hiroki Murakami, Ruri Katsunuma, Kazuo Mishima,

Akira Uno

To check the possibility that greater local activity affects functional connectivity, we make a ‘big’ seed ROI covering brain activity for three kinds of character and illustrate the maps of functional connectivity from the same seed across conditions. We set a bigger brain region that was activated commonly across conditions in the left occipito-temporal cortex as the seed, and calculated functional connectivity from the seed. Three conditions produced similar remote connected brain regions including the basal ganglia, cingulated gyrus, superior frontal gyrus, and inferior frontal gyrus (Fig. S1 and Table S1). Additionally, we could not find connection to the left inferior frontal gyrus in the real *Kanji* character condition. Moreover, pseudo *Kanji* characters did not produce greater extent of connections from the bigger seed to remote brain regions, even though the local activity was still larger at this seed. The results indicate that bigger ROI masked detailed difference of connectivity across different types of characters and might cause false negative results. As well, this result suggests that bigger activity did not always cause larger functional connectivity.

**Fig. S1.** Functional connectivity analyses seeded commonly activated brain region in the left occipito-temporal cortex. Three conditions produced similar remote connected brain regions. The height threshold was *p* < 0.0005 (uncorrected) and the extent threshold was *p* < 0.005 (FDR corrected).

**
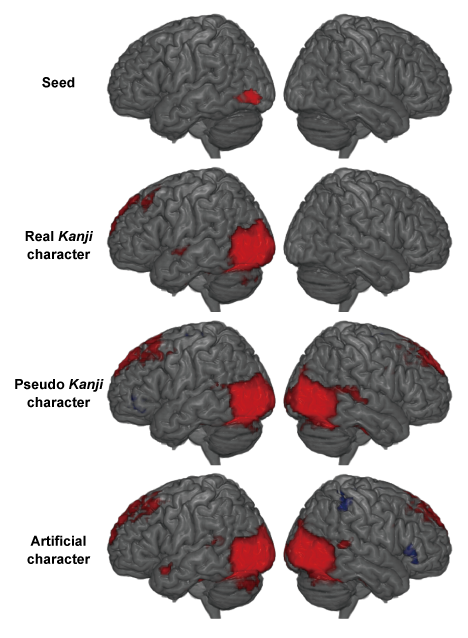
**

**Table S1.** Regions with functional connectivity from the inferior temporal region during observation of the three character types

| Region | MNI coordunate (mm) | | | *T* | voxels |
| --- | --- | --- | --- | --- | --- |
|  | *x* | *Y* | *Z* |  |  |
|  |  |  |  |  |  |
| Real *Kanji* character |  |  |  |  |  |
|  |  |  |  |  |  |
| L superior frontal gyrus | -4 | 58 | 38 | 8.73 | 2042 |
|  |  |  |  |  |  |
| L parahippocampal gyrus | -38 | -22 | -22 | 6.76 | 313 |
|  |  |  |  |  |  |
| R inferior frontal gyrus | 44 | 6 | 30 | 6.41 | 105 |
|  |  |  |  |  |  |
| L middle temporal gyrus | -60 | -6 | -4 | 5.55 | 125 |
|  |  |  |  |  |  |
| R precentral gyrus | 36 | -24 | 38 | -3.69 | 149 |
|  |  |  |  |  |  |
| L precuneus | -12 | -68 | 32 | -3.69 | 1242 |
|  |  |  |  |  |  |
| R cingulate gyrus | 2 | 0 | 38 | -3.69 | 321 |
|  |  |  |  |  |  |
| R sub-gyral | 16 | -8 | 60 | -3.70 | 105 |
|  |  |  |  |  |  |
| L thalamus | -4 | -20 | 10 | -3.70 | 201 |
|  |  |  |  |  |  |
| L superior frontal gyrus | -14 | -6 | 68 | -3.70 | 132 |
|  |  |  |  |  |  |
| Pseudo *Kanji* character |  |  |  |  |  |
|  |  |  |  |  |  |
| L superior frontal gyrus | -8 | 56 | 46 | 7.83 | 3110 |
|  |  |  |  |  |  |
| R inferior frontal gyrus | 46 | 2 | 28 | 5.80 | 132 |
|  |  |  |  |  |  |
| L cingulate gyrus | -6 | -2 | 38 | -3.69 | 718 |
|  |  |  |  |  |  |
| L paracentral lobule | 0 | -34 | 52 | -3.69 | 193 |
|  |  |  |  |  |  |
| R precentral gyrus | 20 | -20 | 64 | -3.69 | 613 |
|  |  |  |  |  |  |
| R thalamus | 4 | -4 | 18 | -3.69 | 627 |
|  |  |  |  |  |  |
| L middle frontal gyrus | -38 | 50 | -4 | -3.69 | 141 |
|  |  |  |  |  |  |
| L caudate | -20 | -2 | 24 | -3.69 | 162 |
|  |  |  |  |  |  |
| Artificial character |  |  |  |  |  |
|  |  |  |  |  |  |
| R middle frontal gyrus | 36 | 34 | 52 | 8.62 | 2900 |
|  |  |  |  |  |  |
| L superior temporal gyrus | -52 | 12 | -18 | 8.16 | 105 |
|  |  |  |  |  |  |
| R inferior frontal gyrus | 48 | 4 | 28 | 7.07 | 120 |
|  |  |  |  |  |  |
| L precuneus | -22 | -60 | 48 | 6.56 | 104 |
|  |  |  |  |  |  |
| R superior temporal gyrus | 58 | -44 | 8 | 5.24 | 146 |
|  |  |  |  |  |  |
| L thalamus | -14 | -8 | 14 | -3.69 | 437 |
|  |  |  |  |  |  |
| R cingulate gyrus | 6 | -26 | 38 | -3.69 | 165 |
|  |  |  |  |  |  |
| R caudate | 22 | -22 | 28 | -3.69 | 120 |
|  |  |  |  |  |  |
| R postcentral gyrus | 26 | -46 | 62 | -3.69 | 142 |
|  |  |  |  |  |  |
| R inferior frontal gyrus | 56 | 22 | 0 | -3.69 | 122 |
|  |  |  |  |  |  |
| R superior frontal gyrus | 20 | -2 | 68 | -3.69 | 238 |
|  |  |  |  |  |  |
| R medial frontal gyrus | 2 | 4 | 46 | -3.69 | 106 |
|  |  |  |  |  |  |
| L thalamus | -10 | -34 | 6 | -3.70 | 434 |
|  |  |  |  |  |  |
| R inferior parietal lobule | 42 | -40 | 60 | -3.70 | 123 |

The height threshold was *p* < 0.0005 (uncorrected) and the extent threshold was *p* < 0.005 (FDR corrected). The auto- and contralaterally-correlated brain regions were omitted.
